# Bioengineering Approaches to In Vitro Modeling of Genetic and Acquired Cardiac Diseases

**DOI:** 10.1007/s11886-025-02218-7

**Published:** 2025-03-20

**Authors:** Linqi Jin, Boeun Hwang, Sarah Rezapourdamanab, Vani Sridhar, Roshni Nandwani, Mehdi Salar Amoli, Vahid Serpooshan

**Affiliations:** 1 Department of Biomedical Engineering, Emory University School of Medicine and Georgia Institute of Technology, Atlanta, GA 30322 USA; 2 Department of Pediatrics, Emory University School of Medicine, Atlanta, GA 30322 USA; 3 Children’s Healthcare of Atlanta, 1075 Haygood Dr., Suite N425, Atlanta, GA 30322 USA; 4 Department of Biology, Emory University, Atlanta, GA 30322 USA

**Keywords:** Cardiac diseases, In vitro model, Human induced pluripotent stem cell (iPSC), Tissue engineering, Heart-on-a-chip, CRISPR/Cas9

## Abstract

**Purpose of Review:**

This review aims to explore recent advancements in bioengineering approaches used in developing and testing in vitro cardiac disease models. It seeks to find out how these tools can address the limitations of traditional in vitro models and be applied to improve our understanding of cardiac disease mechanisms, facilitate preclinical drug screening, and equip the development of personalized therapeutics.

**Recent Findings:**

Human induced pluripotent stem cells have enabled the generation of diverse cardiac cell types and patient-specific models. Techniques like 3D tissue engineering, heart-on-a-chip platforms, biomechanical conditioning, and CRISPR-based gene editing have enabled faithful recreation of complex cardiac microenvironments and disease conditions. These models have advanced the study of both genetic and acquired cardiac disorders.

**Summary:**

Bioengineered in vitro models are transforming the basic science and clinical research in cardiovascular disease by improving the biomimicry and complexity of tissue analogues, increasing throughput and reproducibility of screening platforms, as well as offering patient and disease specificity. Despite challenges in scalability and functional maturity, integrating multiple bioengineering techniques with advanced analytical tools in in vitro modeling platforms holds promise for future precision and personalized medicine and therapeutic innovations.

## Introduction

Cardiovascular diseases are currently the leading cause of illness and death worldwide [1]. With their prevalence continuing to rise, they are projected to contribute to 35.6 million deaths by 2050 worldwide, up from 20.5 million in 2025 [2]. Cardiac diseases can be generally grouped into two categories: genetic and acquired. Genetic cardiac diseases, such as cardiomyopathies, channelopathies, and congenital heart defects (CHDs), are caused by inherited mutations in genes essential for the heart’s structure and function. These mutations can affect proteins involved in muscle contraction or ion channel activity, and disrupt the heart’s electrical and mechanical processes, leading to issues such as arrhythmias, heart failure, and even sudden cardiac death [3]. By contrast, acquired cardiac diseases, including ischemic heart disease, valvular heart disease, inflammatory heart disease, and heart failure, are typically linked to lifestyle factors or environmental triggers and are often developed over time due to poor diet, lack of exercise, or infections [4].

**Fig. 1:**
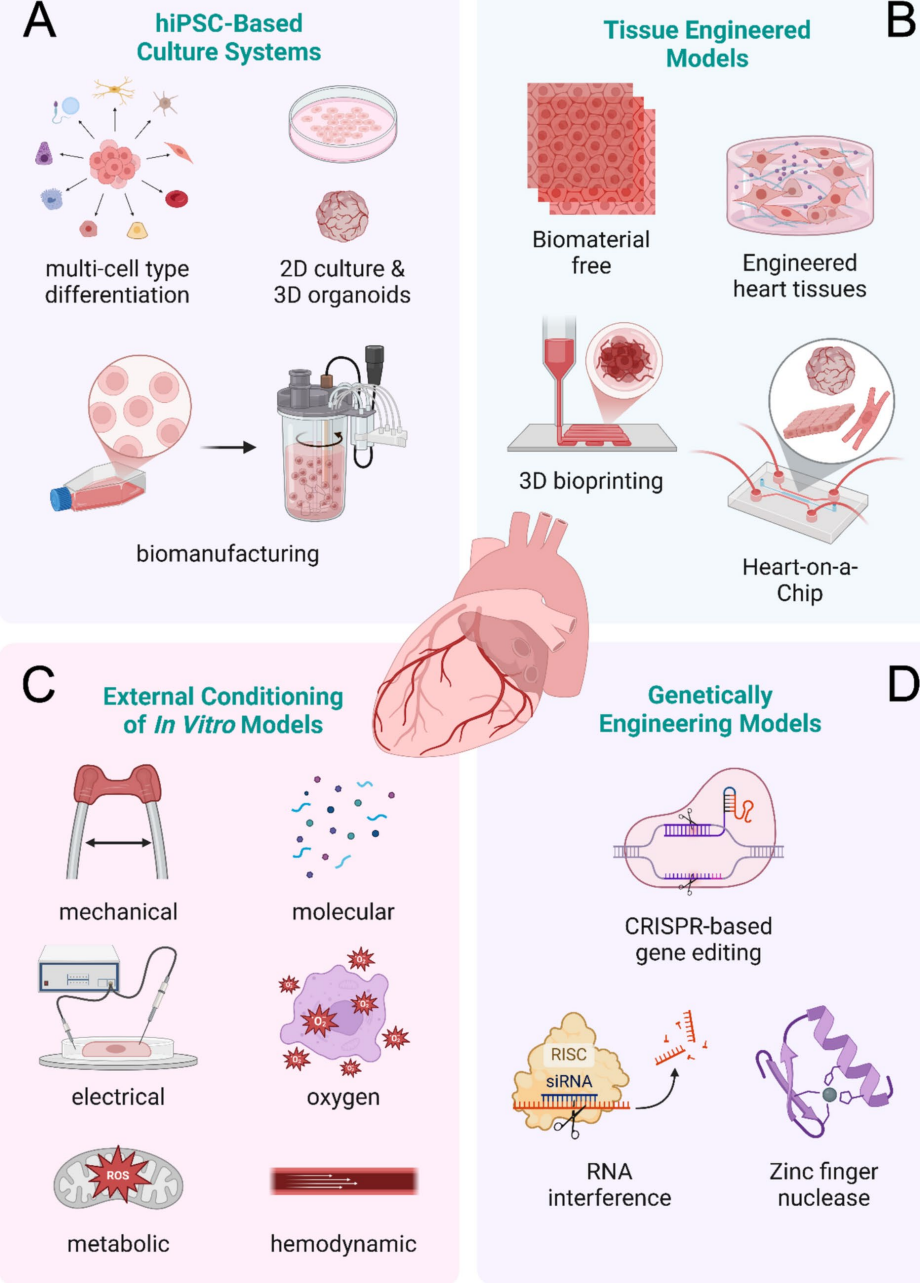
Bioengineering approaches empower advanced in vitro modeling of cardiac diseases. **A** Human induced pluripotent stem cell (hiPSC) technologies enable generation of various patient-specific cardiac cells and their use to model cardiac diseases in 2D and 3D culture platforms. **B** Engineered heart tissue (EHT) models can be fabricated using biomaterial-free or biomaterial-based approaches (*e.g.*, 3D bioprinting) and used to model cardiac disease at higher levels of biomimicry. **C** External conditioning of in vitro cardiac models can be conducted using mechanical, electrical, molecular, or metabolic stressors to faithfully recapitulate cardiac disease pathophysiologies. **D** Genetic engineering can unlock new opportunities for modeling cardiac diseases in vitro, particularly the inherited cardiac disorders. Created in BioRender. Serpooshan, V. (2025) https://BioRender.com/t62h973

Despite advances in clinical research, the development of effective experimental models to simulate cardiac disorders remains challenging [5]. Traditional models provide valuable insights but fail to replicate the complexity of the human heart. For example, mouse models differ significantly from human physiology due to differences in heart rate, ion channel activity, and other species-specific traits [6]. Two-dimensional (2D) cell culture systems, on the other hand, lack the heterogenous, dynamic, and three-dimensional (3D) organization of cellular and extracellular matrix (ECM) components of the native human heart. These limitations hinder accurate prediction of disease progression and therapeutic responses. Bioengineered in vitro models have emerged as a transformative platform to address these gaps, by integrating principles of biology, materials science, engineering, and medicine [7]. Tools and technologies, such as human induced pluripotent stem cells (hiPSCs), 3D tissue engineering, organ-on-a-chip, electro-mechanical conditioning, and genetic engineering, are revolutionizing how cardiac diseases are modeled and studied in vitro [8–11]. These cutting-edge systems not only imitate the structure and function of the native human heart tissue but can also be tailored to reflect the genetic makeup of individual patients [12]. This article reviews current state-of-the-art bioengineering approaches that position in vitro cardiac disease models as powerful tools for understanding disease mechanisms and developing treatment strategies (Fig. 1, Table 1).

**Table 1:** Current In vitro models of genetic and acquired cardiac diseases using different bioengineered approaches

Category	Disease	Platform	Approaches	Study design and finding	Ref
Genetic cardiac diseases	DCM	hiPSC-CMs	CRISPR-based base and prime editing	Corrected the RBM20 mutations; restored splicing and eliminated RNP granule formation in DCM	[86]
	Drug-induced cardiotoxicity	hiPSC-CMs	CRISPRi/a screens	Identified CA12-mediated glycolysis as a contributor to doxorubicin-induced cardiotoxicity and a potential therapeutic target	[87]
	Fabry disease-associated HCM	hESC-CMs	CRISPR/Cas9 gene knockout	Revealed Gb3 accumulation and oxidative stress as contributors to cardiac dysfunction	[83]
	Early HF in HLHS	hiPSC-CMs	Patient-derived hiPSCs	Identified uncompensated mitochondrial oxidative stress as contributors to early HF	[20]
	LQTS	hiPSC-CMs	Patient-derived hiPSCs, CRISPR/Cas9 gene knockout	Corrected LQTS phenotype by gene knockout; validated suppression-replacement gene therapy in patient hiPSC-CMs	[85]
	LQTS	hiPSC-CMs	ZFN	Modeled by overexpression KCNQ1 and KCNH2; treated the model with nifedipine and pinacidil	[84]
	Pregestational diabetes-induced CHDs	hiPSC-COs	hiPSC-COs, metabolic conditioning	Modeled using glucose/insulin modulation; resulted in larger organoids, reduced mitochondria, lipid dysfunction	[40]
	SCN5A overlap syndrome	hiPSC-CMs	Patient-derived hiPSCs	Modeled using patient hiPSC-CMs; evaluated the effects of chronic mexiletine treatment	[21]
	Structural CHDs	ECs	3D bioprinting	3D bioprinted model based on patient-specific echocardiography reconstruction; analyzed the flow hemodynamics and EC function	[61]
Acquired cardiac diseases	Acute cardiac fibrosis	Biomaterial-free microtissues	Biomaterial-free EHTs	Modeled using TGF-β treatment; reversed fibrosis-related changes using BET inhibitors	[47]
	Arrhythmia	hiPSC-CM coculture	hiPSC-CM coculture	Coculture with human adipocytes induced arrhythmic effects via paracrine mechanisms	[38]
	Arrhythmia	hiPSC-derived microtissues	mechanical conditioning	Mechanical stress-induced mutations disrupted calcium dynamics	[74]
	Atrial fibrillation	hiPSC-CM coculture	hiPSC-CM coculture	Coculture with atrial fibroblasts enhanced maturity and modeled AF-like electrophysiological phenotype	[37]
	Cardiac fibrosis	EHTs	Biomaterial-based EHTs	Engineered fibrotic cardiac tissue using PCL nanofibers; observed fibroblast activation and tested Tranilast for antifibrotic effects	[53]
	Cardiac fibrosis	EHTs	EHTs, mechanical conditioning	Cyclic stretch replicated collagen accumulation and stiffness	[73]
	COVID-19 myocarditis	EHTs	EHTs, viral infection	Modeled using hiPSC-CMs, fibroblasts, and macrophages in a collagen-Matrigel matrix; observed cytokine production and sarcomere disassembly	[51]
	COVID-19-induced cardiac dysfunction	hiPSC-COs	hiPSC-COs, molecular conditioning	Cytokine cocktail induced diastolic dysfunction and rescued by BET inhibitors	[77]
	DCM	hiPSC-CMs	Patient-derived hiPSCs	Modeled using hiPSCs with R219H SCN5A variant; observed reduced conduction velocity, delayed calcium handling, prolonged action potential	[35]
	Diabetic cardiomyopathy	hiPSC-CMs	hiPSC-CMs	Recapitulated cellular hypertrophy and lipid accumulation; TGF-β inhibition rescued phenotype	[36]
	Diabetic cardiomyopathy	EHTs	EHTs, molecular conditioning	AGE treatment induced fibrosis, inflammation, and oxidative stress	[79]
	Drug-induced cardiotoxicity	HoCs	EHTs, HoCs, molecular conditioning	Evaluated cardiac response to doxorubicin and dexrazoxane with electrospun scaffolds and microfluidics	[69]
	Endocardial fibroelastosis	Human coronary artery ECs	mechanical conditioning	Stretch-induced EndMT mediated by TGF-β	[72]
	HLHS	EHTs	EHTs, electromechanical conditioning	Pacing revealed reduced contractility and calcium handling defects in HLHS hiPSC-CMs	[34]
	Hypertensive heart disease	HoCs	HoCs, electrical conditioning	Electrical stimulation in hypertrophic hiPSC-CMs modeled chronic hypertension	[75]
	Hypertensive heart disease	EHTs	EHTs, molecular conditioning	modeled pathological gene expression and reduced contractile function by angiotensin II exposure	[76]
	Hypertensive heart disease	EHTs	EHTs, mechanical conditioning	Cyclic stretch induced hypertrophy, increased alignment, and contractility	[71]
	IR injury	H9c2 cardiac myoblasts, neonatal rat and mice CMs	molecular conditioning	Triggered CM ferroptosis by iron overload; identified a novel regulatory p53-Parkin-ACSL4 pathway in hypoxia-reoxygenation injury	[81]
	IR and HFpEF	Biomaterial-free cell sheets	Biomaterial-free cell sheets, oxygen conditioning	Modeled HFpEF by exposing cell sheets to hypoxia and re-oxygenation; assessed contractility and calcium handling	[49]
	IR injury	HoCs	HoC, oxygen conditioning	Observed epicardial contribution to IR injury progression and recovery using oxygen modulation	[67]
	Lipotoxic cardiomyopathy	hiPSC-CMs	hiPSC-CMs, metabolic conditioning	Identified fatty acid oversupply ceramide accumulation as a driver of insulin resistance	[80]
	LQTS and cardiac fibrosis	HoCs	HoCs, electrical and molecular conditioning	Modeled LQTS and fibrosis using electrical pacing and drug administration via microfluidic flow	[68]
	MI	hiPSC-COs	hiPSC-COs, oxygen conditioning	Modeled infarct, border, remote zones of post-MI hearts; JQ1 and doxorubicin tested for drug efficacy	[42]
	MI		3D bioprinting, biomaterial-based EHTs	Modeled post-infarct tissue using aged collagen and cardiac cells	[63]
	MI and IR	hiPSC-COs	hiPSC-COs, oxygen conditioning	Modeled acute MI and fibrosis under hypoxia; observed functional deficits and electrophysiological anomalies	[43]
	Microgravity-induced cardiac dysfunction	HoCs	HoCs, microgravity conditioning	Revealed oxidative stress and mitochondrial dysfunction in spaceflight models	[70]
	Oxidative stress-induced cardiac dysfunction	H9c2 cardiac myoblasts, neonatal rat ventricular myocytes, and hiPSC-CMs	oxidative stress conditioning	Modeled using hydrogen peroxide injury; ghrelin expression protected against oxidative stress-induced damage	[78]
	Tachycardia-induced cardiomyopathy	EHTs	tachpacing, metabolic, and oxygen conditioning	Tachypaced EHTs showed hypoxia, increased glucose metabolization, and contractile dysfunction	[50]

*DCM* dilated cardiomyopathy, *hiPSC* human induced pluripotent stem cell, *CM* cardiomyocyte, *HCM* hypertrophic cardiomyopathy, *HF* heart failure, *HLHS* hypoplastic left heart syndrome, *hESC* human embryonic stem cell, *LQTS* long QT syndrome, *ZFN* zinc finger nuclease, *hiPSC-CO* hiPSC-cardiac organoids, *CHD* congenital heart disease, EC endothelial cell, *EHT* engineered heart tissue, *BET* bromodomain and extraterminal family inhibitor, *AF* atrial fibrillation, *PCL* polycaprolactone, *AGE* advanced glycation end-products, *HoC* heart-on-a-chip, *HFpEF* heart failure with preserved ejection fraction, *MI* myocardial infarction, *IR* ischemia – reperfusion

## hiPSC-Based Models of Cardiac Disease

The advancement of hiPSC technologies has enabled the generation of a variety of human- and patient- specific cardiac cells, including cardiomyocytes (CMs), cardiac fibroblasts (CFs), endothelial cells (ECs), smooth muscle cells (SMCs), pericytes, and immune cells [13–18]. Precise modulation of key signaling pathways, such as Wnt/β-catenin, BMP, and Activin/Nodal, guides mesodermal progenitors toward a cardiac fate and subsequently into distinct cardiac cell subtypes [19]. This process mirrors the natural stages of heart development, making hiPSC-based cellular models highly attractive for studying genetic cardiac diseases, especially those manifesting during early developmental stages (Fig. 1A). For example, Xu et al. studied early heart failure (HF) using hiPSC-derived CMs (hiPSC-CMs) from patients with hypoplastic left heart syndrome (HLHS) and found its association with uncompensated mitochondrial oxidative stress [20]. Nasilli et al. established a model of SCN5A overlap syndrome using hiPSC-CMs with the SCN5A-1795insD mutation, which exhibited both features of Brugada syndrome and cardiac conduction disease [21]. They further evaluated the therapeutic effect of chronic mexiletine treatment on the disease. Despite these significant advancements, immaturity and lack of scalability remain as some key challenges, hindering the clinical relevance and large-scale use of hiPSC-derived cardiac cells in disease modeling. Continuous progress has been made in improving the production and maturation of these cells, as well as advancing the automation of their differentiation processes. Buikema et al. reported a method to enable the in vitro expansion of hiPSC-CMs without altering their contractile function using GSK-3β inhibition and contact removal [22]. Recently, a study by Deogharia et al. showed promise in enhancing hiPSC-CM maturity using histone lysine demethylase KDM5 inhibition, which epigenetically upregulated the expressions of oxidative phosphorylation, fatty acid oxidation, and sarcomere genes and enhanced myofibril organization and mitochondrial function [23]. Moreover, Kriedemann et al. and Prondzynski et al*.* described protocols for generating hiPSC-CMs using stirred suspension systems with less batch variability and more resource efficiency as compared to standard monolayer CM differentiation [24, 25]. Moving forward, as each cardiac cell type plays a critical role in heart development and function, substantial efforts are being dedicated to generating region- and function- specific cardiac cell subtypes from hiPSCs, equipping the recapitulation of specific cardiac disease mechanisms that involve multicellular interplay. For example, Mikryukov et al*.* and Liu et al*.* demonstrated the use of BMP10 signaling pathway to generate hiPSC-endocardial cells with enhanced expression of NKX2-5 and NRG1 as compared to hiPSC-ECs differentiated via BMP4 modulation [26, 27]. These cells exhibited the ability to undergo endothelial-to-mesenchymal transition (EndMT) and form heart valvular interstitial cells, showing potential for modeling endocardial-related CHDs and valvular heart disease. The differentiation of the right ventricular, outflow tract, and the pacemaker sinoatrial node-like CMs from hiPSCs was also reported by Wiesinger et al. and others via the tuning of Nodal and retinoic acid signaling [28–31].

### 2D hiPSC-Based In Vitro Models

Given their advantages in simplicity and scalability, 2D hiPSC-based cell cultures are the most widely used platforms for the in vitro research of cardiac disease (Fig. 1A, Table 1) [32]. Monocultures of hiPSC-CMs have been integrated with advanced measurement and analytical tools like microelectrode arrays (MEAs), patch clamp, and single-cell RNA sequencing (scRNA-seq), for high-throughput screening of disease phenotypes and drug responses [33, 34]. In one study, Djemai et al. generated hiPSC-CMs from patients with dilated cardiomyopathy (DCM) carrying the R219H SCN5A variant [35]. Significantly reduced conduction velocity, prolonged action potential duration, and delayed calcium transient uptake, reuptake, and duration were detected in patient-derived CMs compared to control CMs. Similarly, in the context of acquired cardiac diseases, Tang et al. were able to recapitulate the pathological features of diabetic cardiomyopathy, including cellular hypertrophy, lipid accumulation, and increased susceptibility to high-glucose/high-lipid challenge in hiPSC-CMs derived from patients with type 2 diabetes mellitus (T2DM) [36]. The hypertrophic phenotype was further rescued in T2DM hiPSC-CMs by TGF-β inhibition, underscoring the model’s efficacy for investigating molecular mechanisms and evaluating therapeutic compounds.

Taking a step further, coculture systems of hiPSC-CMs with non-CM cells have been developed to provide paracrine signals, mechanical support, and vasculature, for more physiologically relevant disease modeling. For example, Brown et al. cocultured hiPSC-derived atrial CMs with atrial fibroblasts on a patterned surface and reported significantly enhanced structural, electrical, contractile, and metabolic maturation of CMs after 6 weeks compared to monoculture [37]. The patterned coculture displayed closer modeling of atrial fibrillation (AF)-like electrophysiological phenotype and greater sensitivity for detecting drug efficacy than monoculture. In another study by Morrissette-McAlmon et al., human adipocytes cocultured with hiPSC-CMs demonstrated proarrhythmic effects through paracrine mechanisms, suggesting a pathogenic role of infiltrating human adipocytes on myocardial tissue [38].

### 3D hiPSC-Based Cardiac Organoids

Derived from the codifferentiation and self-organization of multiple hiPSC-derived cardiac cells, 3D hiPSC-cardiac organoids (hiPSC-COs) show promise in replicating the spatial composition, structure, and function of the native heart, presenting as a valuable platform for modeling heart chamber defects and other intricate cardiac diseases (Fig. 1A, Table 1) [39]. In a study by Lewis-Israeli et al., key characteristics of the heart, such as internal chamber structures, multi-lineage cardiac cell types, and vascular networks were recapitulated in the self-assembling hiPSC-COs [40]. Pregestational diabetes-induced CHDs were modeled with the modulation of glucose and insulin concentrations in the differentiation media, resulting in increased organoid size, reduced mitochondrial content, dysfunctional lipid metabolism, and impaired structural organization. In a follow-up study, Volmert et al. induced anterior–posterior patterning in the hiPSC-COs using an endogenous gradient of retinoic acid [41]. The patterned organoids were treated with ondansetron, a common prescription medication for pregnancy-related nausea, also known as an hERG sodium channel blocker, and exhibited diseased electrophysiological properties linked to ventricular cardiac defects, including reduced contractile frequency and amplitude, and prolonged action potential duration. Given their multicellular composition and 3D geometries, hiPSC-COs have been also employed to model acquired cardiac diseases such as myocardial infarction (MI). In a study by Richards et al., oxygen-diffusion gradient and chronic adrenergic stimulation were incorporated into hiPSC-COs to recreate the infarct, border, and remote zones of post-MI hearts [42]. Pathological metabolism, fibrosis, and calcium handling related to organoid infarction injury were observed at transcriptomic, cellular, and tissue levels with a non-genetic pathological basis. An antifibrotic agent (JQ1) and a chemotherapy drug (doxorubicin) were further tested in this MI model which demonstrated its potential for HF drug testing and drug-induced cardiotoxicity screening. More recently, Song et al. modeled the phenotypes of acute MI and cardiac fibrosis by subjecting hiPSC-COs to hypoxia-induced ischemia and ischemia–reperfusion (IR) injury, resembling features including cardiac cell death, functional deficits, collagen deposition, disrupted calcium ion handling, and electrophysiological anomalies [43].

While highly promising, current hiPSC-based 2D cellular models and 3D hiPSC-CO systems are still compromised by limited tissue complexity, maturity, and reproducibility, as well as small (mm scale) dimensions, unable to fully replace in vivo studies, especially for multifaced cardiac disease modeling and preclinical therapeutic testing. The continuous emergence of tissue engineering and integrative analytical tools, however, is rapidly advancing the design optimization and disease modeling applications of 3D hiPSC-CO platforms [44].

## Engineered Heart Tissues (EHTs)

### Biomaterial-free 3D models

As the native heart tissue exhibits highly compact cellular organization and intricate cell–cell interactions, substantial efforts have been focused on fabricating EHTs that closely replicate the native cardiac tissue microenvironment. Biomaterial-free manufacturing approaches, such as cardiac spheroids and cell sheets, have been adapted to achieve high cell density and robust intercellular communication without the use of synthetic or natural scaffolds (Fig. 1B, Table 1) [45, 46]. Biomaterial-free EHTs are typically created by coculturing various cardiac cells, including CMs, ECs, and fibroblasts, which are either pre-differentiated or derived from primary cell sources, allowing them to self-assemble into functional structures. A recent study by Reyat et al. demonstrated the generation of chamber-specific cardiac microtissues and their use in modeling acute cardiac fibrosis via TGF-β treatment [47]. The chamber-specific microtissues were fabricated using either ventricular or atrial CMs combined with ECs and fibroblasts. Features including myofibroblast activation, increased collagen deposition, and impaired calcium handling, were observed upon TGF-β treatment, evidencing the successful induction of cardiac fibrosis. Through the administration of TGF-β receptor inhibitor and bromodomain and extraterminal family inhibitor (BETi), these pathological changes were reversed, demonstrating the potential of these cardiac microtissues for pharmacological screening. Additionally, cell sheets have been developed to model human myocardium, leveraging temperature-sensitive culture surfaces [48]. In a study by Yamasaki et al., cardiac cell sheets were used to model IR injury and assess its effects on myocardial contractile function [49]. The cardiac cell sheets were exposed to hypoxia (1% O_2_) and re-oxygenation (20% O_2_) conditions and assessed for contractility, calcium transients, and sarcomeric structures. While many parameters, such as calcium transient magnitude and contractile force, recovered after re-oxygenation, the maximum relaxation velocity remained significantly reduced, indicating an underlying diastolic dysfunction, which demonstrated the potential use of the conditioned cell sheets as a model of heart failure with preserved ejection fraction (HFpEF).

### Biomaterial-Based Tissue Engineered 3D Models

Another crucial component in fabricating EHTs is the biomaterial scaffold. A variety of hydrogels have been used to generate EHTs, including collagen, fibronectin, fibrin, Matrigel, and decellularized extracellular matrix (dECM) (Fig. 1A, Table 1). Since the ECM microenvironment plays a key role in directing cellular responses, the recreation of a proper 3D environment is essential for functional EHTs. Furthermore, the addition of ECM biomaterials enhances the structural stability of the EHTs, enabling facilitated handling and mechanical stimulation of the tissues. Biomaterial-based EHTs can be subjected to biochemical and physical cues to simulate different cardiac diseases. For example, tachycardia-induced cardiomyopathy was modeled in a study by tachypacing 3D EHTs around two silicone posts [50]. The EHTs were fabricated by casting hiPSC-CMs into molds with fibrinogen, Matrigel, and thrombin, and subsequently pacing the constructs at a minimum of 3 Hz to recapitulate tachycardia-induced cardiac dysfunction. Using these tachypaced EHTs, the study revealed that tachycardia results in contractile dysfunction through increased hypoxia and glucose metabolization. Another study by Bailey et al. modeled COVID-19 myocarditis using EHTs containing hiPSC-CMs, fibroblasts, and macrophages seeded in a collagen-Matrigel matrix between 2 polydimethylsiloxane (PDMS) posts [51]. Cytokine production, sarcomere disassembly, and cell death were demonstrated in the EHTs, indicating myocardial dysfunction caused by SARS-COV-2 infection of hiPSC-CMs, despite the resistance of ECs, macrophages, and fibroblasts to the virus.

Cardiac diseases can disrupt the homeostasis of the tissue microenvironment, leading to dynamic changes in the cellular or ECM compositions and tissue mechanical properties [52]. Therefore, the scaffolding biomaterials of EHTs can be fine-tuned to mimic the diseased myocardium. For example, Ruocco et al. engineered the tissue architecture, ECM composition, and stiffness of EHTs to model cardiac fibrosis and induce the activation of CFs into myofibroblasts [53]. The fibrotic cardiac tissue was generated using electrospun polycaprolactone (PCL) nanofibers functionalized with collagen type I and fibronectin at a 7:3 ratio. The electrospun PCL fibers provided topographical and mechanical similarity, while the collagen-to-fibronectin ratio mimicked the ECM composition of fibrotic heart tissue. This study showed that approximately 80% of fibroblasts can undergo activation on the ECM-coated PCL scaffold with and without TGF-β stimulation. Moreover, treating the cardiac fibrosis model with Tranilast, an antifibrotic drug, reduced fibrosis-related characteristics, such as α-SMA expression and collagen I and fibronectin production, which showcased the responsiveness of this model to established treatments.

### 3D Bioprinted Models of Cardiac Disease

As a robust additive tissue biomanufacturing approach, 3D bioprinting has enabled the fabrication of cardiac tissue analogues at a higher degree of structural and cellular complexity by allowing precise deposition of biomaterials, living cells, and biological molecules (Fig. 1B) [54]. In the past few years, this technology has evolved to encompass extrusion, inkjet, and laser-based bioprinting modalities [55, 56]. A wide variety of bioinks, composed of natural and synthetic polymers, along with cells, such as CMs, SMCs, CFs, and ECs, have been utilized to bioprint 3D cardiac constructs, capturing the physiological properties of the native tissue for disease modeling [57]. For example, hiPSC-CMs encapsulated in extrusion bioprinted constructs were able to preserve their elongated cell morphology and functionality while maintaining high structural stability in a study by Lappi et al. [58]. Similarly, enhanced maturation, indicated by sarcomere structure and gene expression, was observed in hiPSC-CMs within 3D bioprinted tissues compared to 2D culture as shown by Wolfe et al. [59].

Moreover, the incorporation of medical imaging data, such as magnetic resonance imaging (MRI) and computed tomography (CT), into 3D bioprinting workflows has provided a unique capacity for recreating and analyzing patient- and disease- specific cardiac architectures in vitro, including chambered structures and tissue anomalies [60]. This facilitates the study of cardiac disease pathophysiology, particularly those involving geometry-dependent cell-ECM and cell–cell interactions. For example, Cetnar et al. employed digital light processing (DLP) bioprinting to construct anatomically accurate models of the developing human heart based on patient-specific fetal echocardiography data [61]. Flow hemodynamic patterns within constructs were examined using ultrasound and 4D MRI techniques and analyzed for their effects on EC growth and function. In another study, Kupfer et al. bioprinted a multi-chambered heart analogue based on a human heart MRI dataset [62]. They utilized the freeform reversible embedding of suspended hydrogels (FRESH) technique to fabricate EHTs with macrotissue level functionality. An optimized bioink containing hiPSCs, gelatin methacrylate (GelMA), collagen methacrylate (ColMA), fibronectin, and laminin was used. Within the chambered constructs, hiPSCs were effectively differentiated into CMs, CFs, and ECs in situ, exhibiting contiguous electromechanical properties and pumping function. In terms of acquired cardiac diseases, Basara et al. utilized aged type I collagen and aged hiPSC-derived cardiac cells to bioprint an aged post-MI myocardial tissue model. Multi-region constructs closely replicated the biomechanical and biochemical characteristics of post-infarct tissue and served as a valuable platform for analyzing stem cell-derived extracellular vesicle treatment on modulating the scar tissue microenvironment [63].

## Heart-On-A-Chip (HoC) Platforms

In addition to the cellular and structural complexity of EHTs, the inclusion of dynamic microenvironmental components, such as flow velocity, pressure, shear stress, electrical signals, and biochemical gradients, is also critical for modeling cardiac diseases in a controllable manner. To this end, heart-on-a-chip (HoC) systems have gained significant attention as a versatile platform, integrating principles of both tissue engineering and microfluidics (Fig. 1B, Table 1) [10]. HoC devices typically consist of a microfluidic chip, cardiac cells, ECM-like hydrogels, actuators, and sensors [64]. Microfluidic chips are often made from silicon or PDMS using micro- or nano- fabrication techniques, such as photolithography. Culture media flow and other external stimuli, such as electrical or mechanical conditioning, can be simultaneously applied to the cardiac tissues in the HoCs. Outputs such as contractile motion, fluorescence, and electrophysiological signals, can be recorded using biosensors or cameras, enabling the evaluation of cellular behavior in response to these stimuli [65, 66]. For example, Bannerman et al. developed a biowire-based HoC containing both epicardial and myocardial tissues to model IR injury by modulating the oxygen level in perfusion [67]. Retention and migration of epicardial cells, along with preservation of cardiac viability and function, were observed in the post-IR HoCs, highlighting the epicardial contribution to the IR injury progression and recovery. In another study by Min et al., an HoC system composed of EHTs and microfluidic chips was employed to model long QT syndrome (LQTS) and cardiac fibrosis [68]. Electrical pacing was applied to assess the abnormalities in LQTS-derived EHTs, while the pro-fibrotic agent TGF-β and the anti-fibrotic drug losartan were administrated via microfluidic flow to model the induction and treatment of cardiac fibrosis. Furthermore, HoC systems have been equipped with multi-material scaffolds and sensory systems to improve their functionality and broaden their range of applications. Liu et al. created an anisotropic HoC by combining 3D printed, electrospun scaffolds and microfluidic chips [69]. Cardiac tissue response to the chemotherapeutic agent doxorubicin and the cardioprotective agent dexrazoxane was evaluated in this model, demonstrating its suitability for testing the toxicity of different therapeutic compounds on cardiac tissues. Similarly, Mair et al. designed an HoC platform to assess the impact of spaceflight on cardiac function [70]. A hybrid scaffold composed of dECM and reduced graphene oxide was used to generate EHTs with enhanced maturity and electroconductivity. Giant magnetoresistive sensors were incorporated for real-time automated contractile force measurements. Mitochondrial dysfunction and oxidative stress were revealed to be key regulators of cardiac dysfunction in microgravity exposed HoCs.

## External Conditioning of Cardiac Tissue Models

To faithfully recapitulate cardiac disease pathophysiologies, external in vitro conditioning of EHTs has been employed, using mechanical, electrical, molecular, or metabolic stressors (Fig. 1C). Mechanical conditioning involves subjecting cardiac cells or tissues to forces like cyclic stretch or compression, replicating the mechanical loads they experience under pathological conditions. For example, Ruan et al. showed that EHTs exposed to mechanical stretch exhibit increased cell alignment, CM hypertrophy, and enhanced contractility, replicating the features of hypertensive heart disease [71]. Vorisek et al. reported stretch-induced EndMT in human coronary artery ECs, indicating TGF-β mediated formation of endocardial fibroelastosis [72]. Coeyman et al. used a bioreactor platform capable of simultaneous mechanical loading and live mechanical analyses to evaluate cell-mediated remodeling of 3D tissue constructs composed of murine CFs within a fibrin matrix under cyclic stretch [73]. Mechanical stress-mediated CF activation, characterized by collagen accumulation and increased tissue stiffness, was effectively captured in this model, replicating the phenotype of cardiac fibrosis. Additionally, Guo et al. showed that mechanical stress-induced mutations in hiPSC-derived microtissue models can disrupt calcium dynamics, contributing to arrhythmogenic events [74].

Electrophysiological properties of cardiac tissue are closely linked to the heart function and serve as key indicators of cardiac diseases. Therefore, electrical conditioning is widely used for in vitro modeling and induction of stress conditions. For example, Zhao et al. performed chronic electrical conditioning on hiPSC-CMs obtained from patients with left ventricle hypertrophy using an engineered biowire platform [75]. The stimulation frequency was gradually increased from 2 to 6 Hz to mimic chronic increased workloads arising from hypertension and then maintained at 3 Hz for up to 8 months. Long-term electrical stimulation resulted in distinct differences in hypertrophy-associated gene expression and contractile function between hypertrophic and healthy hiPSC-CMs. This is while no differences in cell viability and CM content were found, highlighting the importance of electrical conditioning in modeling polygenic cardiac diseases in chronic settings. Similarly, electromechanical stress (1 Hz pacing, 1 mN diastolic preload) was applied to HLHS-derived EHTs for over 24 days in a study by Krane et al. [34]. The HLHS EHTs exhibited significantly reduced contractile force, impaired responsiveness to high stimulation frequencies, a gradual decline in electrically responsive CM numbers, and intrinsic calcium handling defects. These findings collectively revealed that impaired maturation in HLHS hiPSC-CMs limits their ability to respond to growth cues.

Other approaches such as molecular and metabolic conditioning also aid the recapitulation of pathological cardiac conditions. Horton et al. utilized angiotensin II exposure to recapitulate hypertensive conditions in EHTs, resulting in pathological gene expression, increased occurrences of early after depolarization events, and decreased contractile function [76]. Mills et al. performed a rapid screening of cytokine combinations using phosphoproteomics and single nuclei RNA sequencing to model the COVID-19-induced cytokine storm and cardiac dysfunction in hiPSC-COs [77]. A cocktail of interferon gamma, interleukin 1β, and poly(I:C) was identified to induce diastolic dysfunction in hiPSC-COs. The disease phenotype was recovered by treatment with BETi, resulting in decreased expression of viral response genes and ACE2, and reduced SARS-CoV-2 CM infection. Furthermore, Kok et al., developed an oxidative stress model using hydrogen peroxide injured H9c2 cardiac myoblasts, neonatal rat ventricular myocytes, and hiPSC-CMs, which revealed the cardioprotective effect of ghrelin expression against oxidative stress [78]. Wang et al. treated murine-based EHT models with multiple dosages of advanced glycation end-products (AGEs), which led to the stimulation of several markers of fibrosis, inflammation, and oxidative stress, replicating the key features of diabetic cardiomyopathy [79]. Bekhite et al. investigated the role of ceramide accumulation in lipotoxic cardiomyopathy by exposing hiPSC-CMs to fatty acid oversupply, mimicking diabetic conditions [80]. The study identified ceramide accumulation as a key driver of insulin resistance, oxidative stress, increased auto/mitophagy, and mitochondrial dysfunction, suggesting de novo ceramide synthesis modulation as a potential therapeutic target for metabolic cardiomyopathy. Additionally, Xiao et al. treated rat CMs with iron overload and identified a novel regulatory p53-Parkin-ACSL4 pathway underlying CM ferroptosis in hypoxia-reoxygenation (IR)-induced cardiomyopathy [81].

## Genetically Engineered In Vitro Models of Cardiac Disease

As a powerful tool for modulating gene expression in hiPSCs and other cell lines, genetic engineering has unlocked new opportunities for modeling cardiac diseases in vitro, especially inherited disorders (Fig. 1D) [82]. Techniques such as CRISPR-based gene editing, zinc finger nuclease (ZFN), and RNA interference (RNAi) enable precise gene knock-ins and knockouts to introduce or correct mutations, as well as genome-wide interference/activation screens (CRISPRi/a) of disease-causing genes. These methods have been instrumental in creating disease models and elucidating gene functions and pathways implicated in cardiac disorders.

For example, Song et al. utilized CRISPR/Cas9 to knock out the GLA gene and created a human embryonic stem cell (hESC)-derived model of Fabry disease (FD)-associated hypertrophic cardiomyopathy [83]. The resulting cardiac dysfunction, caused by GLA deficiency, included globotriaosylceramide (Gb3) accumulation, disrupted Rab GTPase-mediated vesicle trafficking, impaired autophagy, and increased reactive oxygen species. Similarly, Wang et al. developed an LQTS model by overexpressing dominant negative mutations in the ion channel genes KCNQ1 and KCNH2 in iPSC-CMs using ZFN [84]. Characteristic LQTS phenotypes, including prolonged action potential duration, were recapitulated in the model and were later alleviated by the addition of nifedipine (L-type calcium channel blocker) or pinacidil (KATP-channel opener). Conversely, Dotzler et al. performed isogenic CRISPR/Cas9 correction of KCNQ1 in type 1 LQTS (LQT1) hiPSC-CMs, which demonstrated normalized levels of action potential duration and served as a positive control for testing KCNQ1-SupRep (suppression-and-replacement) gene therapy in LQT1 hiPSC-CMs [85]. Nishiyama et al. knocked out RBM20 mutations in hiPSC-CMs by adenine base editing and prime editing to correct DCM, which normalized alternative splicing of cardiac genes, restored RBM20 nuclear localization, and eliminated RNP granule formation [86]. For applications other than disease phenotype alteration, Liu et al. implemented CRISPRi/a bidirectional pooled screens to identify causative genes in doxorubicin-induced cardiotoxicity in hiPSC-CM models and identified CA12-mediated glycolysis as a previously unreported contributor and therapeutic target [87]. Chirikian et al. generated three fluorescent reporter hiPSC lines by targeting fluorescent reporter constructs to highly-conserved, chamber-specific genes using CRISPR/Cas9, which allowed for the isolation of chamber-specific hiPSC-CMs, facilitating potential lineage tracing studies in hiPSC-based models of CHDs [88].

## Conclusions and Future Directions

Integrating multiple bioengineering approaches into one in vitro platform has emerged as a promising approach to enhance the physiological relevance of in vitro modeling systems and transform the study of cardiac diseases and their therapies. Preclinically, these cardiac disease models hold great promise for high-throughput drug testing and personalized therapeutic innovation. However, challenges such as limited maturity, complexity, scalability, and reproducibility continue to limit their predictive accuracy. Future efforts should be invested in addressing these hurdles and unlocking the full potential of these models. The advent of advanced tissue biomanufacturing technologies, and 3D bioprinting in particular, has markedly accelerated the pace of interdisciplinary research efforts, enabling the creation of highly complex and heterogenous, patient-specific models of cardiac disease. Further, the incorporation of advanced analytical tools, such as spatial multi-omics, real-time imaging, and electrophysiology monitoring, should be considered to increase the resolution of disease mechanism studies using these platforms. By tapping into these robust tools, bioengineered in vitro models are poised to lead disease mechanism discoveries, advance precision medicine, and ultimately improve outcomes for patients with cardiac diseases.

## Key References

- Tu C, Caudal A, Liu Y, Gorgodze N, Zhang H, Lam C, et al. Tachycardia-induced metabolic rewiring as a driver of contractile dysfunction. NATURE BIOMEDICAL ENGINEERING. 2024;8(4).
  - ○ Findings from this study suggest that hiPSC-based EHTs can be combined with multiple external stress conditioning for studying metabolic rewiring in tachycardia-induced cardiomyopathy.
- Mair DB, Tsui JH, Higashi T, Koenig P, Dong Z, Chen JF, et al. Spaceflight-induced contractile and mitochondrial dysfunction in an automated heart-on-a-chip platform. Proceedings of the National Academy of Sciences. 2024;121(40):e2404644121.
  - ○ Findings from this study suggest that automated heart-on-a-chip platform facilitates cardiac disease modeling under rare and extreme conditions.
- Nishiyama T, Zhang Y, Cui M, Li H, Sanchez-Ortiz E, McAnally JR, et al. Precise genomic editing of pathogenic mutations in RBM20 rescues dilated cardiomyopathy. Sci Transl Med. 2022;14(672):eade1633.
  - ○ Findings from this study suggest the efficacy of CRISPR/Cas9-based editing in rescuing DCM in hiPSC-CM models.

## Data Availability

No datasets were generated or analysed during the current study.
